# Genome Sequence of an Alphabaculovirus Isolated from *Choristoneura murinana*

**DOI:** 10.1128/genomeA.01135-13

**Published:** 2014-01-30

**Authors:** George F. Rohrmann, Martin A. Erlandson, David A. Theilmann

**Affiliations:** a Department of Microbiology, Oregon State University, Corvallis, Oregon, USA; b Saskatoon Research Centre, Agriculture and Agri-Food Canada, Saskatoon, SK, Canada; c Pacific Agri-Food Research Centre, Agriculture and Agri-Food Canada, Summerland, BC, Canada

## Abstract

The genome sequence of a baculovirus from *Choristoneura murinana* is 124,689 bp, with a G+C content of 50%, and contains 148 putative open reading frames. The virus is a member of the group I alphabaculoviruses and is most closely related to several other viruses that infect *Choristoneura* species.

## GENOME ANNOUNCEMENT

The European fir budworm *Choristoneura murinana* (Lepidoptera, Tortricidae) is infected by a nucleopolyhedrovirus (NPV) (ChmuNPV) (1). ChmuNPV has a relatively wide host range and infects several additional tortricids, including the codling moth, *Cydia pomonella*, and at least one noctuid, *Mamestra brassicae*. During infection, it produces spindle-shaped cytoplasmic occlusions (1), possibly derived from gp37 (ac64) (2). It was characterized by restriction endonuclease digestion and shown to be distinct from the NPVs of *Choristoneura occidentalis* (ChocNPV) and *Choristoneura fumiferana* (CfMNPV) (western and eastern spruce budworms, respectively) (3). Subsequently, this was confirmed by the PCR amplification and sequencing of a portion of the lef-8 gene (4). To further characterize this virus, the complete sequence of its genome was determined using 454 sequencing technology and found to be 124,689 bp and to encode 148 predicted proteins of over 50 amino acids. A total of 143 of the open reading frames (ORFs) showed similarity to other baculovirus genes in the database, including all 37 core genes. ChmuNPV encodes the fusion protein gp64 and is a member of the group I alphabaculoviruses.

The relatedness of baculovirus genomes is normally determined by combining PIF2 and LEF8 amino acid sequences. ChmuNPV PIF2 is unusual in that it has an N-terminal extension of 126 amino acids that is not closely related to anything in the database and is not found in other baculoviruses. However, when the extension was removed and the combined PIF2-LEF8 sequences were compared, ChmuNPV was closest to CfMNPV and ChocNPVs, in a lineage distinct from *Cydia  rosanus* NPV (ChroNPV), *Orgyia pseudotsugata* MNPV (OpMNPV), and *Hyphantria cunea* NPV (HycuNPV), which were the other most closely related viruses to ChmuNPV. All the branch points showed bootstrap levels of 99% or 100% and amino acid identities of 93% (OpMNPV) and 94% (HycuNPV), to over 97% (Cf-, Choc-, and ChroNPVs).

More extensive comparison of about 40 predicted proteins showed that they were on average about 91% identical to those from CfMNPV, with some ORFs (those for polyhedrin and ubiquitin) being 99% identical (5). However, other ORFs showed lower identity, such as those for Ac66 (66%) and vp80 (Ac104) (72%). The genome (124.7 kb) is about 4 kb smaller than that of the close relative ChocNPV (6). Part of this is due to the lack of a viral enhancing factor (vef) gene that could account for 2.3 kb of the difference. The ChmuNPV genome showed a pattern of homologous regions similar to those of ChocNPV and ChroNPV (6).

Although most of the genome was assembled from the set of 454 sequence reads (138× coverage), a region within the desmoplakin gene (*ac66*) did not link up. To resolve this gap, primers were designed to span the region and PCR amplification resulted in two amplicons. Upon sequencing the two products, we found the difference to be in the number of repeats of a sequence within Ac66. The longer ORF that was used in the assembly reported here had three repeats of a 75-nucleotide (nt) sequence, whereas the smaller ORF had only two repeats. This suggested that the sample that was sequenced was a mixture of two variants.

### Nucleotide sequence accession number.

The genome sequence of ChmuNPV has been deposited in GenBank under the accession no. KF894742.

## References

[1] Naser WL Harvey JP Huger AM Huber J 1987 *Choristoneura murinana* nuclear polyhedrosis virus: comparative biochemical and biological examination of replication in vivo and in vitro. J. Gen. Virol. 68:1251–1260. 10.1099/0022-1317-68-5-1251

[2] Rohrmann GF 2011 Baculovirus molecular biology. National Library of Medicine, Bethesda, MD.

[3] Rohrmann GF Melgaard S Beaudreau GS 1982 Characterization of DNA from three nuclear polyhedrosis viruses pathogenic for *Choristoneura* sp. J. Invertebr. Pathol. 40:237–241. 10.1016/0022-2011(82)90121-5

[4] Rohrmann GF 2013 Characterization of baculoviruses from the Martignoni collection. J. Invertebr. Pathol. 114:61–64. 10.1016/j.jip.2013.04.005 23628143

[5] de Jong JG Lauzon HA Dominy C Poloumienko A Carstens EB Arif BM Krell PJ 2005 Analysis of the *Choristoneura fumiferana* nucleopolyhedrovirus genome. J. Gen. Virol. 86:929–943. 10.1099/vir.0.80490-0 15784887

[6] Thumbi DK Béliveau C Cusson M Lapointe R Lucarotti CJ 2013 Comparative genome sequence analysis of *Choristoneura occidentalis* Freeman and *C*. *rosaceana* Harris (*Lepidoptera*: *Tortricidae*) alphabaculoviruses. PLoS One 8:e68968. 10.1371/journal.pone.0068968 23861954 PMC3702617

